# Pericytes Contribute to the Disruption of the Cerebral Endothelial Barrier via Increasing VEGF Expression: Implications for Stroke

**DOI:** 10.1371/journal.pone.0124362

**Published:** 2015-04-17

**Authors:** Ying Bai, Xinjian Zhu, Jie Chao, Yuan Zhang, Cheng Qian, Peicheng Li, Dongfang Liu, Bing Han, Lei Zhao, Jianqiong Zhang, Shilpa Buch, Gaojun Teng, Gang Hu, Honghong Yao

**Affiliations:** 1 Department of Pharmacology, Medical School of Southeast University, Nanjing, China; 2 Department of Physiology, Medical School of Southeast University, Nanjing, China; 3 Department of Radiology, Medical School of Southeast University, Nanjing, China; 4 Department of Immunology, Medical School of Southeast University, Nanjing, China; 5 Department of Pharmacology and Experimental Neuroscience, University of Nebraska Medical Center, Omaha, Nebraska, United States of America; 6 Department of Pharmacology, Nanjing Medical University, Nanjing, China; Center for Interdisciplinary Research in Biology (CIRB), Collège de France / CNRS / INSERM, FRANCE

## Abstract

Disruption of the blood-brain barrier (BBB) integrity occurring during the early onset of stroke is not only a consequence of, but also contributes to the further progression of stroke. Although it has been well documented that brain microvascular endothelial cells and astrocytes play a critical role in the maintenance of BBB integrity, pericytes, sandwiched between endothelial cells and astrocytes, remain poorly studied in the pathogenesis of stroke. Our findings demonstrated that treatment of human brain microvascular pericytes with sodium cyanide (NaCN) and glucose deprivation resulted in increased expression of vascular endothelial growth factor (VEGF) via the activation of tyrosine kinase Src, with downstream activation of mitogen activated protein kinase and PI3K/Akt pathways and subsequent translocation of NF-κB into the nucleus. Conditioned medium from NaCN-treated pericytes led to increased permeability of endothelial cells, and this effect was significantly inhibited by VEGF-neutralizing antibody. The *in vivo* relevance of these findings was further corroborated in the stroke model of mice wherein the mice, demonstrated disruption of the BBB integrity and concomitant increase in the expression of VEGF in the brain tissue as well as in the isolated microvessel. These findings thus suggest the role of pericyte-derived VEGF in modulating increased permeability of BBB during stroke. Understanding the regulation of VEGF expression could open new avenues for the development of potential therapeutic targets for stroke and other neurological disease.

## Introduction

The blood-brain barrier (BBB) is a dynamic network for maintenance of the CNS homeostasis by restricting the passage of toxic substances into the brain parenchyma. Nutrients such as glucose and amino acids cross the BBB using specific transporters [1,2]. Peptides, in general, poorly cross the BBB [3,4], but can be transported into the brain via specific transporters expressed in brain endothelium under physiological or pathological conditions [5,6]. BBB is mainly composed of brain microvascular endothelial cells (BMECs), astrocytic endfeet, pericytes and neurons that are critical for maintenance of homeostasis in the central nervous system [7–9]. In addition to BMECs and astrocytes, pericytes play a vital role in regulation of BBB integrity through communication with BMECs via the release of soluble factors such as TGF-β [10–12]. Moreover, it has been demonstrated that pericytes are necessary for the formation of BBB during embryogenesis and that loss of pericytes leads to compromised BBB integrity as shown in the pericyte-deficient animal model, suggesting thereby an important role of pericytes in the maintenance of BBB integrity [13,14].

BBB dysfunction has been demonstrated in various neurological disorders including stroke, Alzheimer's disease as well as epilepsy [15–17]. Moreover, disruption of BBB integrity is not only a common consequence of, but also contributes to the progression of stroke [18]. Extensive studies have documented that BMECs and astrocytes play critical roles in the maintenance of BBB integrity [19–21]. However, the role of pericytes in stroke remains largely unknown.

Ischemic damage to BBB has been extensively studied in cellular and animal models of stroke [22,23]. One study demonstrated that MMP-9 derived from pericytes induced migration of these cells from the endothelium, leading to loss of pericytes and ensuing BBB damage [24]. Interestingly, similar to glial activation by inflammatory agents, pericytes have also been shown to be activated in response to lipopolysaccharide leading release of cytokines that further exacerbate neuroinflammation [25]. Interestingly, previous study demonstrated that a chronic BBB opening caused by pericyte loss and/or degeneration leads to neuronal uptake of multiple blood-derived neurotoxic products as well as reductions in microcirculation and capillary flow causing tissue hypoxia that in turn results in a chronic neuronal dysfunction and degenerative changes [26]. While the role of pericytes in stroke has been gaining attention since capillary pericytes are rapidly lost following cerebral ischemia in both experimental animal as well as in human stroke [27], the detailed mechanism(s) underlying how ischemia mediates pericytes to contribute to BBB damage, has largely remained elusive.

Previous studies reported that vascular endothelial growth factor (VEGF) is one of the potent mediators of the BBB breach as evidenced by the fact that VEGF increased BBB permeability by decreasing the expression of tight junction protein claudin-5 [21,28]. A recent study has reported that VEGF derived from astrocytes was involved in endothelial barrier disruption [29], further confirming the critical role of VEGF in the BBB disruption in the context of stroke. Based on the finding that sodium cyanide (NaCN), a general metabolic inhibitor, induces chemical hypoxia in an *in vitro* stroke model [30], we sought to examine the effect of this agent on the expression of VEGF in pericytes and the subsequent effect on the BBB integrity in stroke.

The present study was aimed at exploring the mechanism(s) by which NaCN mediates the induction of VEGF in primary human pericytes and its functional implications in stroke. Understanding the regulation of VEGF expression and its functional relevance could provide insights into the development of therapeutic strategies aimed at restoring the BBB breach in stroke patients.

## Method and Materials

### Animals and focal stroke model

Adult male C57BL/6J mice (20.0–25.5 g, 8–10 weeks old) were used in this study. All animals were housed under conditions of constant temperature and humidity on a 12-hour light/12-hour dark cycle, with lights on at 7:00 AM. Food and water were available *ad libitum*. Animals were anesthetized by an overdose of isoflurane followed by pneumothorax before perfusion. All animal procedures were performed in strict accordance with the ARRIVE guidelines and animal protocols approved by the Institutional Animal Care and Use Committee of the Medical School of Southeast University.

### Reagents

Neutralizing VEGF (MAB 293) antibody and IgG were purchased from R&D Systems (Minneapolis, MN, USA). The specific PI3-kinase inhibitor LY294002, MEK1/2 inhibitor U0126 or PD98059, p38 inhibitor SB 203580, JNK inhibitor SP600125, Src kinase inhibitor inhibitor-PP2 were obtained from Calbiochem (San Diego, CA). Iκκ-2 inhibitor SC514 was ordered from Sigma Chemicals (St. Louis, MO, USA). The concentrations of these inhibitors were based on our previous reports [31,32].

### Focal stroke model

Briefly, as reported earlier [33,34], stroke was generated by making an incision between the right orbit and the right external auditory canal under a stereoscopic microscope. The scalp and temporalis muscle were exposed and the zygomatic arch was snipped to expose the proximal section of the right middle cerebral artery (MCA). In order to effectively occlude the right MCA, immediately after intravenous injection of a photosensitizer Rose Bengal solution (100mg/kg, 10mg/ml in normal saline; Sigma-Aldrich) through the tail vein, photoillumination with green laser (wavelength 532nm, GL532TA-100FC, Shanghai Laser & Optics Century) was performed on the MCA for 2mins by using a 100μm optic fiber connected to a laser diode controller (power at 35, ADR-1805, Shanghai Laser & Optics Century). Sham operation was performed with the same surgical procedures but with injection of the PBS instead. The mice were then allowed to awaken and returned to their cages. Total surgery lasted for up to 20mins. There was no surgery-related mortality noted.

### Cell culture

Primary human brain vascular pericytes were purchased from ScienCell (Carlsbad, CA, USA) and cultured in the pericyte medium (provided by ScienCell). Cell culture dishes were coated with poly-L-lysine (2μg/cm^2^) and were used in passages 2–5. Human brain microvascular endothelial cells (HBMECs) were obtained also from ScienCell and cultured in endothelial medium (provided by ScienCell) and were used for passages 4–14.

### Glucose deprivation

To mimic the ischemic condition *in vitro*, human brain pericytes were exposed to glucose-deprived medium according a previous study [30]. Briefly, the glucose-free Locke's medium was comprised of 154mM NaCl, 5.6mM KCl, 2.3mM CaCl_2_, 1mM MgCl_2_, 3.6mM NaHCO_3_, 5mM HEPES, pH7.2, supplemented with gentamicin (5 mg/L). Cultured pericytes were incubated with glucose-free Locke's medium for 6 hours, 12 hours and 24 hours. Control cultures were incubated in Locke's buffer containing 10mM glucose.

### Cytokine/Chemokine analysis by Luminex

Supernatants collected from treated and untreated pericytes were monitored for cytokines/chemokines using a MilliplexTM MAP kit (Human Cytokine/Chemokine Magnetic Bead Panel) from Millipore according to the manufacturer’s instructions and our previous study [35,36]. Expression of cytokines/chemokines was repeated three separate times.

### Western blotting

Pericytes exposed to different treatments were harvested and total protein was extracted using the Mammalian Cell Lysis kit (Sigma, St. Louis, MO). Nuclear lysates were isolated using the NE-PER Nuclear and Cytoplasmic Extraction kit (Pierce, Rockford, IL). Equal amounts of the proteins were run on a sodium dodecyl sulfate-polyacrylamide gel (12%) under reducing conditions. The proteins were then transferred to PVDF membranes and were blocked with 5% non-fat dry milk in PBS. The membranes were then incubated with antibodies recognizing VEGF (Santa Cruz Biotechnology, Dallas, TX, 1:200), the phosphorylated forms of ERK, JNK, p38 and Akt (Cell Signaling, Danvers, MA 1:200), NF-κB p65 (Cell Signaling, 1:1000). Following washing three times, the membranes were then incubated with goat anti-mouse/rabbit secondary antibodies conjugated with horseradish peroxidase (1:5000). Signals were detected by chemiluminescence and imaged on the Microchemi 4.2 (DNR, Israel) digital image scanner according to our previous studies [31,32,37]. Quantification was performed by densitometry using Image J software (NIH).

### Permeability of endothelial barrier

According to our previous study [37], primary HBMECs were plated onto Transwell inserts (0.4μm pore size). The cells were grown for 5 days to achieve confluence followed by culturing the cells in conditioned media from pericyte treated with NaCN in the presence of either neutralizing VEGF antibody (500ng/ml) or an IgG isotype control antibody (as a negative control). Twenty four hours later, 200μl of FITC-conjugated Dextran-4 (1mg/ml; Sigma) was added to the upper chamber of the transwell plates to detect the changes in monolayer permeability. After 30mins, aliquots (100μl) were collected from the lower chamber for fluorescent measurement using excitation and emission wavelengths of 480 and 530nm respectively (Biotek Synergy H1 multimode microplate reader instrument).

### Isolation of brain microvessels

Mice were separated into the sham and the stroke groups (n = 8). Twenty four hours after surgery, mice were perfused under anesthesia and the brains were removed and immediately immersed in ice-cold isolation buffer A according to our previous study [31].

### Immunofluorescence staining

Isolated brain microvessels were then smeared on ploy-L-lysine coated glass slides followed by fixation with 4% formaldehyde for 20mins at RT. The slides were then rinsed with PBS, permeabilized with 0.1% Triton X-100 for 30mins and blocked in immunoblocking buffer (10% normal goat serum, 0.3% Triton X-100 in PBS) for 2 hours at RT. Samples were then incubated with mouse VEGF antibodies (Proteintech, Chicago, IL, 1:50) and rabbit PDGF-β receptor antibodies (Cell Signaling,1:200), overnight at 4°C. The slides were washed and incubated with AlexaFluor 488-conjugated anti-mouse and AlexaFluor 594-conjugated anti-rabbit immunoglobulin G (IgG) (Invitrogen, Grand Island, NY) and mounted with the mounting medium (Prolong Gold Anti-fade Reagent; Invitrogen, Grand Island, NY, USA). Fluorescent images were acquired using Olympus FV 1000 microscope and were analyzed with Image J software. Average fluorescence intensities of VEGF and PDGF-β receptor were calculated using Image J by analysis of total 80 images for one group taken from 10 individual microvessels isolated from each mouse brain, 8 mice/group. Values are reported as average intensity above background±SD.

### *In vivo* magnetic resonance imaging (MRI)

Based upon our previous study [38], we have successfully developed an effective method for the PEGylation of superparamagnetic iron oxide nanoparticles (SPIONs). The diameter of the nanoparticles is in the range of 8–12nm. The hydrodynamic diameter of the nanoparticles was measured to be 25±3.6nm with a polydispersity index of 0.26 by dynamic light scattering (DLS) in aqueous medium. A saline solution of SPIONs-PEG was injected into mice via the tail vein at 0.5 hour after stroke operation at a dose normalized to be 10mg Fe/kg bodyweight. MRI was carried out on a 7.0 Tesla small animal magnetic resonance system (Bruker PharmaScan, Germany) as described previously [39]. *In vivo* T2-weighted images were performed 24 hours post-stroke following injection of SPIONs, which was used to examine the BBB integrity using a two-dimensional turbo spin-echo sequence (repetition time/echo time = 2,000/50ms). Twelve axial slices with a slice thickness of 1mm, a field of view of 20×20mm and a matrix of 256×256 were positioned over the brain excluding the olfactory bulb. Quantitative image analysis was performed by investigators blinded to the experimental groups. The signal noise ratios (SNR) of both ipsilateral and contralateral hemisphere were calculated using Paravision 5.0 software with the following equation: the average signal intensity (SI)/the background noise (SD). Results were presented as ratio of the SNRs between ipsilateral and contralateral hemisphere.

### Assessment of the BBB Integrity

According our previous studies [31,37], BBB integrity was performed in C57BL/6 mice. C57BL/6 mice were divided into 4 groups (n = 6): (a) Sham, (b) Stroke, (c) Stroke plus anti-VEGF neutralizing antibody, (d) Stroke plus isotype control antibody. In the antibody plus stroke group, antibody was first injected (i.p.) for two days at a concentration of 25μg/mouse/injection (anti-VEGF neutralizing antibody; or IgG isotype control antibody; R&D systems) followed by stroke operation on day 3. Twenty four hours later, animals were injected in the tail vein with 200μl Evans blue (2%, 4ml/kg; Sigma) in PBS that was allowed to circulate for 40mins. The mice were then anesthetized with isoflurane in oxygen and perfused with 30ml heparinized saline through the left ventricle. The brains were then harvested and homogenized in PBS (1:10 g/v). The homogenates were precipitated in 15% trichloroacetic acid (1:1 v/v) and centrifuged at 1,000g for 10mins. The pH was adjusted by adding 125μl of 5M sodium hydroxide to 500μl supernatant aliquots. Evans blue was measured spectrophotometrically at 620nm.

### Statistical analysis

Statistical analysis was performed using SigmaPlot software (SigmaPlot 11.0). Data are presented as means±SD. Significance was established using a t test for paired values. Intergroup comparisons were performed using one-way ANOVA, with the Bonferroni correction for multiple comparisons.

## Results

### NaCN-mediated up-regulation of VEGF expression in primary pericytes

**Fig 1:**
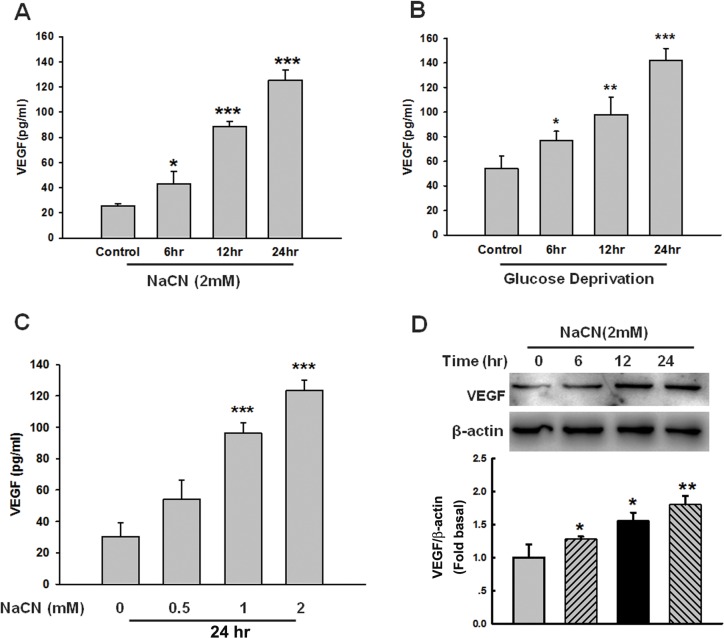
NaCN mediated up-regulation of VEGF in primary human pericytes. **(A)** NaCN mediated induction of VEGF expression in primary human pericytes. Cells were incubated with NaCN (2mM) for different time points (6, 12 and 24 hours), followed by collection of media for assay of VEGF expression by Luminex assay. **(B)** Pericytes were exposed to glucose-deprived culture media for 6, 12 and 24 hours followed by Luminex assay for detection of VEGF. **(C)** Dose curve of NaCN on the expression of VEGF in pericytes by Luminex assay. **(D)** Effect of NaCN on the expression of VEGF in primary human pericyte by western Blot. All the data are presented as mean±SD of three individual experiments (n = 3). *p<0.05, **p<0.01, ***p<0.001 vs control group.

Previous study has demonstrated that NaCN (an inhibitor of mitochondrial respiration used to mimic hypoxia) plays a critical role in the pathogenesis of stroke [30,40]. However, whether NaCN induces activation of pericytes and the mechanisms involved in this process, remain largely unknown. In order to examine the effect of NaCN on the release of cytokines/chemokines from pericytes, supernatants from NaCN-treated pericytes was subjected to human cytokine antibody array analysis. Treatment of pericytes with NaCN resulted in dramatic induction of VEGF expression using Luminex assay as shown in Fig 1A as well as in Table 1. To validate the effect of NaCN on the expression of VEGF in pericytes, we cultured pericytes under conditions of glucose deprivation to mimic stroke and assessed the expression of VEGF. As shown in Fig 1B, similar to NaCN exposure, glucose deprivation also resulted in up-regulation of VEGF protein expression. We next examined the concentration curve of NaCN mediated up-regulation of VEGF in pericytes. Pericytes were treated with different concentrations of NaCN (0.5, 1 and 2mM) for 24 hours and monitored for expression of VEGF protein by Luminex analysis. As shown in Fig 1C, NaCN induced a significant increase in VEGF expression at a concentration of 1mM and 2mM. We next determined the optimal time of NaCN-mediated up-regulation of VEGF in pericytes. Primary human pericytes were exposed to NaCN (2mM) at varying time points and assessed for VEGF expression by western blotting. As shown in Fig 1D, there was a time-dependent increase of VEGF expression following NaCN treatment, with the maximal expression of VEGF observed at 24 hours after NaCN treatment. These data thus demonstrated that NaCN treatment resulted in increased expression of VEGF in primary human pericytes.

**Table 1:** Effect of NaCN on the expression of cytokines/chemokines in human brain pericytes by Luminex assay (Mean, n = 3) *-: not detectable.

Cytokine (pg/ml)	Control	6hr	12hr	24hr
**G-CSF**	3.23	9.47	5.97	11.40
**Eotaxin**	2.24	2.95	2.24	2.25
**GM-CSF**	26.77	34.40	20.10	31.90
**VEGF**	**25.73**	**51.73**	**88.47**	**125.33**
**IFN-γ**	-*	**-**	**-**	**-**
**IL-1α**	5.37	8.17	5.93	7.47
**IL-1β**	7.05	8.85	8.85	10.51
**IL-2**	1.03	1.90	1.65	1.67
**IL-4**	**-**	**-**	**-**	**-**
**IL-3**	1.05	1.23	1.23	1.07
**IL-5**	13.80	14.87	13.30	13.33
**IL-6**	369.00	796.33	815.67	965.67
**IL-7**	1.43	1.97	1.30	1.67
**IL-9**	14.73	26.50	21.30	27.80
**IL-10**	4.33	5.73	4.77	5.40
**IL-12p40**	**-**	**-**	**-**	**-**
**IL-12 p70**	2.83	4.00	2.90	3.60
**LIF**	7.33	11.37	10.80	14.40
**IL-13**	35.90	54.60	47.40	47.43
**Lix**	491.67	426.67	384.67	278.00
**IL-15**	3.20	7.10	3.63	4.63
**IL-17**	1.10	1.15	1.23	1.15
**IP-10**	60.77	57.90	43.83	51.33
**KC**	2424.33	2868.00	2332.00	2650.67
**MCP-1**	3167.00	3152.00	2648.00	2149.00
**MIP-1α**	2.80	4.63	3.10	3.73
**MCSF**	10.40	13.53	11.33	11.33
**MIP2**	20.60	24.30	18.60	25.83
**MIG**	7.27	8.00	8.73	8.00
**Rantes**	29.49	28.12	22.04	22.60
**TNF-α**	2.09	3.04	2.71	2.55

### NaCN-mediated expression of VEGF involves Src activation

**Fig 2:**
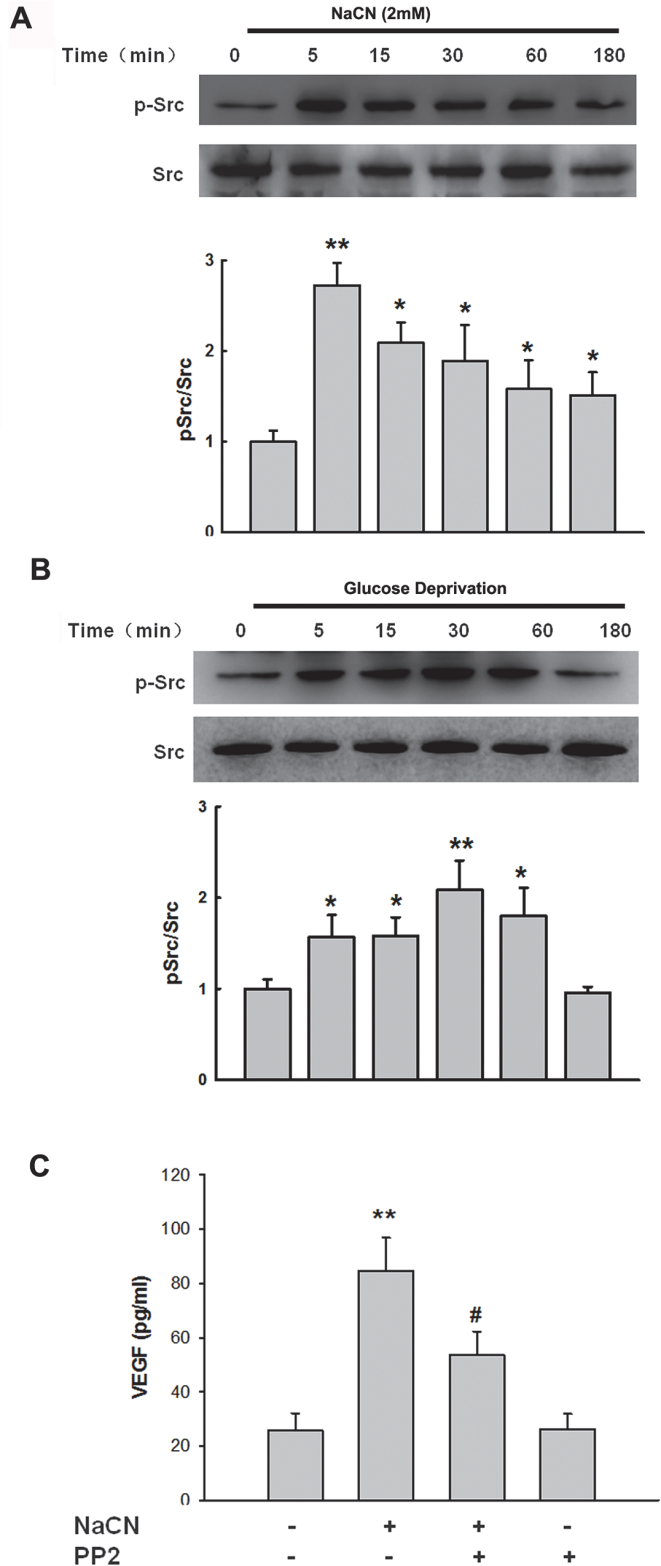
NaCN-mediated induction of VEGF involves Src kinase activation. **(A)** NaCN (2mM) induced Src phosphorylation in a time-dependent manner in primary human pericytes. Representative immunoblots from four separate experiments are presented. **(B)** Exposure of pericytes to glucose deprivation induced Src phosphorylation in a time-dependent manner in primary human pericytes. Representative immunoblots from four separate experiments are presented. **(C)** Inhibition of Src activity by Src inhibitor-PP2 (10μM) resulted in amelioration of NaCN-mediated induction of VEGF. All the data are presented as mean±SD of four individual experiments (n = 4). *p<0.05, **p<0.01 vs control group; #p<0.05 vs NaCN-treated group.

Role of tyrosine kinase Src has been shown to be involved in expression of VEGF during focal cerebral ischemia reperfusion in rats [41]. Moreover, mounting evidence from other studies also demonstrates that activation of Src kinase plays a critical role in VEGF expression [42,43]. We thus wanted to examine whether Src activation also played a role in NaCN-mediated induction of VEGF. As shown in Fig 2A, treatment of primary human pericytes with NaCN resulted in increased phosphorylation of Src in a time-dependent manner, with the peak activation at 5mins and a gradual decrease thereafter. Consistent with the effect induced by NaCN, exposure of pericytes to glucose deprivation resulted in Src phosphorylation as shown in Fig 2B. To confirm the role of Src activation in NaCN mediated induction of VEGF, pericytes were pre-treated with the Src inhibitor-PP2 for 1hour followed by incubation with NaCN for 24 hours. As shown in Fig 2C, treatment of cells with the Src inhibitor PP2 significantly inhibited NaCN-induced production of VEGF.

### NaCN-mediated activation of Src involves activation of MAPKs and PI3K/Akt pathways

**Fig 3:**
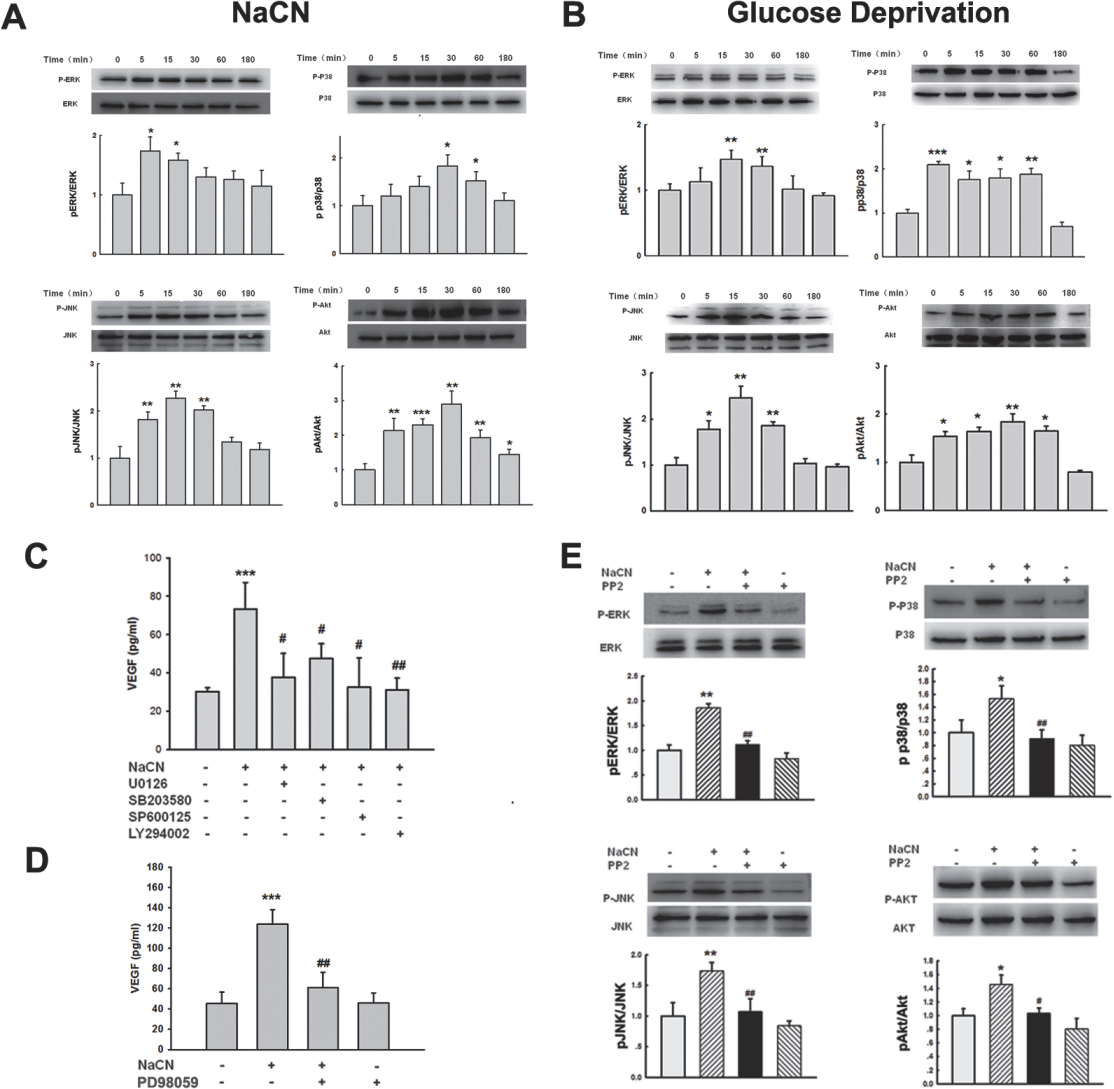
NaCN-mediated induction of VEGF expression involves MAPKs and PI3K/Akt cell signaling pathways. **(A)** Western blot analysis of time-dependent activation ERK, p38, JNK MAPKs MAPKs and PI3K/Akt pathways by NaCN (2mM) in primary human pericytes. **(B)** Glucose deprivation resulted in activation ERK, p38, JNK MAPKs and PI3K/Akt pathways in primary human pericytes. **(C)** Inhibition of the ERK, p38, JNK MAPKs and Akt pathways by MEK1/2 (U0126, 10μM), p38 inhibitor (SB203580, 10μM), JNK inhibitor (SP600125, 10μM) and PI3K inhibitor (LY294002, 5μM) resulted in amelioration of NaCN-mediated induction of VEGF. **(D)** Pretreatment of pericytes with another MEK inhibitor-PD98059 (10μM) significantly inhibited NaCN-mediated induction of VEGF. **(E)** Pretreatment of primary human pericytes with Src inhibitor-PP2 resulted in inhibition of NaCN-mediated phosphorylation of ERK, p38, JNK and Akt pathways. Representative immunoblots and densitometric analyses of pERK/ERK, pp38/p38, pJNK/JNK and pAkt/Akt from 4 separate experiments are presented. All the data are indicated as mean±SD of four individual experiments (n = 4). *p<0.05; **p<0.01, ***p<0.001 vs control group; #p<0.05; ##p<0.01 vs NaCN-treated group.

Given that MAPK kinase and PI3K/Akt pathways play critical roles in expression of VEGF [43,44], we next sought to examine whether these pathways contributed to NaCN-mediated induction of VEGF. As shown in Fig 3A, treatment of primary human pericytes with NaCN resulted in increased time-dependent phosphorylation of ERK, p38, JNK and Akt. Further validation of this finding was performed by exposure of pericytes to glucose deprivation. As shown in Fig 3B, glucose deprivation resulted in phosphorylation of ERK, p38, JNK in pericytes. In order to examine the role of these pathways in NaCN-mediated up-regulation of VEGF, primary human pericytes were pretreated with inhibitors specific for the respective signaling pathways prior to stimulation with NaCN and assessed for expression of VEGF. As shown in Fig 3C, pretreatment of cells with MEK (U0126, 10μM), p38 (SB203580, 10μM), JNK (SP600125, 10μM) and PI3K (LY294002, 5μM) inhibitors significantly decreased NaCN-mediated induction of VEGF. The functional role of NaCN-induced ERK activation in mediating VEGF expression was also corroborated using another MEK inhibitor-PD98059 (Fig 3D). These findings thus underpinned the role of ERK, p38, JNK MAPKs and PI3K/Akt in NaCN-mediated induction of VEGF in primary human pericytes.

Having determined that Src and MAPKs and PI3K/Akt pathways play critical roles in the induction of VEGF, we next dissected the link between Src activation and MAPKs as well as PI3K/Akt pathways. Pericytes pretreated with the Src inhibitor PP2 were exposed to NaCN, followed by assessment of activation of the MAPKs/Akt pathways. As shown in Fig 3E, pretreatment of pericytes with PP2 significantly inhibited the phosphorylation of MAPKs (ERK, p38 and JNK,) and Akt pathways induced by NaCN, suggesting thereby that Src lies upstream of MAPKs and PI3K/Akt.

### Involvement of NF-kB in NaCN-mediated expression of VEGF in primary human pericytes

**Fig 4:**
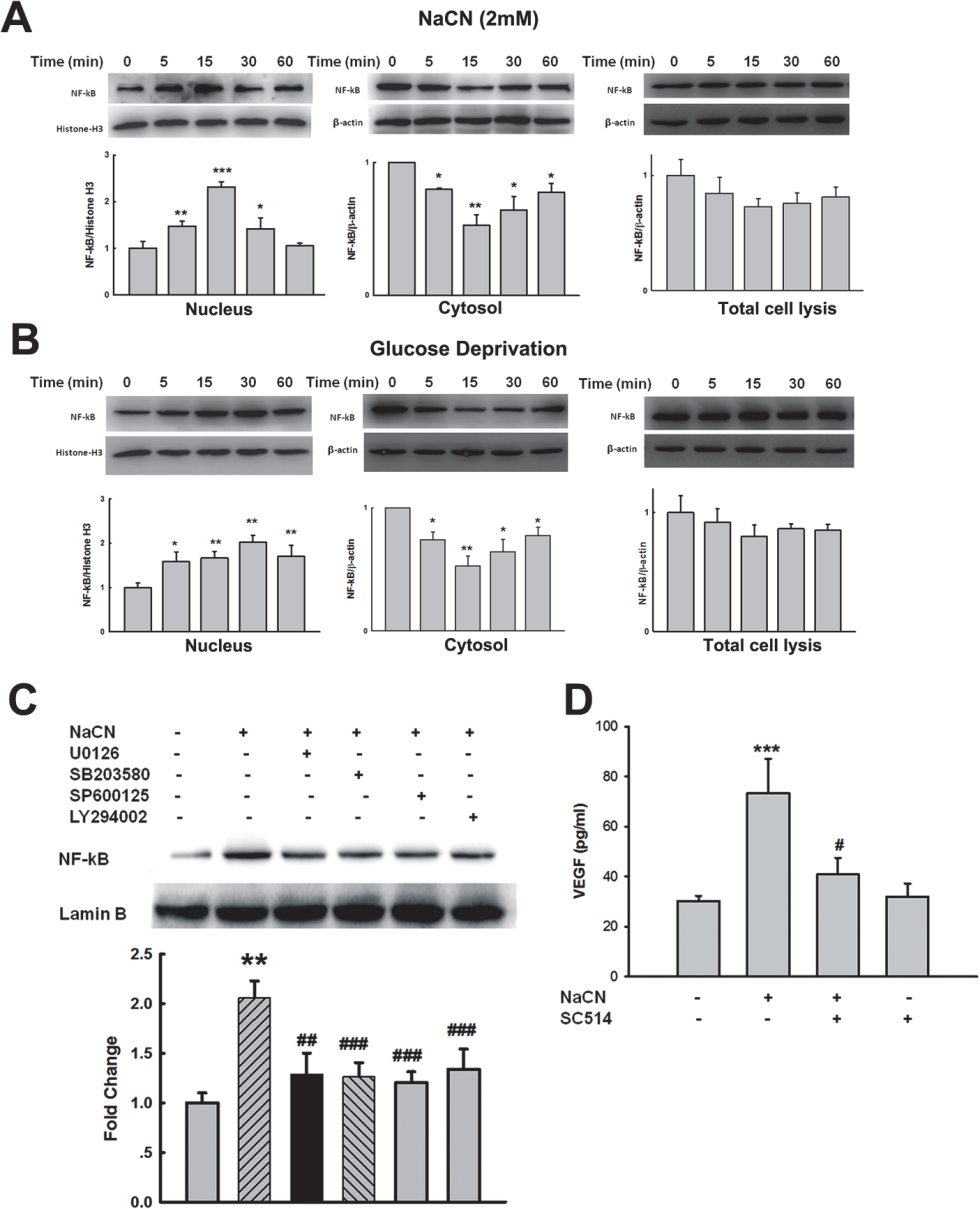
NaCN-mediated induction of VEGF expression involves NF-κB activation. **(A)** Treatment of primary human pericytes with NaCN (2mM) resulted in time-dependent increase in translocation of the p65 subunit of NF-κB into the nuclear fraction (right panel) with concomitant decrease in the cytosolic fraction (middle panel). NaCN failed to exert significant effect on the expression of NF-kB in the total cell lysis from pericytes. **(B)** Exposure of primary human pericytes to glucose deprivation resulted in time-dependent increase in translocation of the p65 subunit of NF-κB into the nuclear fraction (right panel) with concomitant decrease in the cytosolic fraction (middle panel). NaCN failed to exert significant effect on the expression of NF-kB in the total cell lysis from pericytes. **(C)** Pretreatment of primary human pericytes with MEK1/2 (U0126, 10μM), p38 inhibitor (SB203580, 10μM), JNK inhibitor (SP600125, 10μM) and PI3K inhibitor (LY294002, 5μM) resulted in inhibition of NaCN-mediated NF-kB translocation of the p65 subunit of NF-κB into the nucleus. **(D)** Pretreatment with the Ikk2 inhibitor-SC514 (5μM) resulted in inhibition of NaCN-mediated induction of VEGF. All the data are mean±SD of four individual experiments (n = 4). *p<0.05; **p<0.01;***p<0.001 vs control group; #p<0.05; ##p<0.01, ###p<0.001 vs NaCN-treated group.

Based on a previous report demonstrating activation of NF-kB pathway in the induction of VEGF [45], we next determined whether NF-kB pathway was also involved in NaCN-mediated induction of VEGF expression in primary human pericytes. As shown in Fig 4A, NaCN treatment of pericytes resulted in a rapid translocation of NF-kB p65 into the nucleus with concomitant reduction in the cytoplasmic level of NF-kB. However, NaCN treatment failed to exert significant effect on the expression of NF-kB in the total cell lysis from pericytes (Fig 4A). These findings were further confirmed in pericytes cultured in glucose deprivation condition as shown in Fig 4B. We next wanted to examine whether there existed a link between activation of ERK, p38, JNK MAPKs, and PI3K/Akt with NF-kB. Primary human pericytes were pretreated with ERK, p38, JNK or PI3K inhibitor(s) followed by treatment with NaCN and assessed for translocation of NF-kB. As shown in Fig 4C, ERK, p38, JNK and PI3K inhibitors significantly inhibited NaCN-mediated activation of NF-kB. Further validation of the role of NF-κB p65 in NaCN-mediated expression of VEGF was carried out by pretreating the cells with the Ikk-2 inhibitor-SC514 and assessing for induction of VEGF. As shown in Fig 4D, pretreatment of cells with SC514 (5μM) resulted in amelioration of NaCN-mediated induction of VEGF, thereby underscoring the involvement of NF-κB p65 in this process.

### Disruption of endothelial integrity by NaCN-treated pericytes involves VEGF

**Fig 5:**
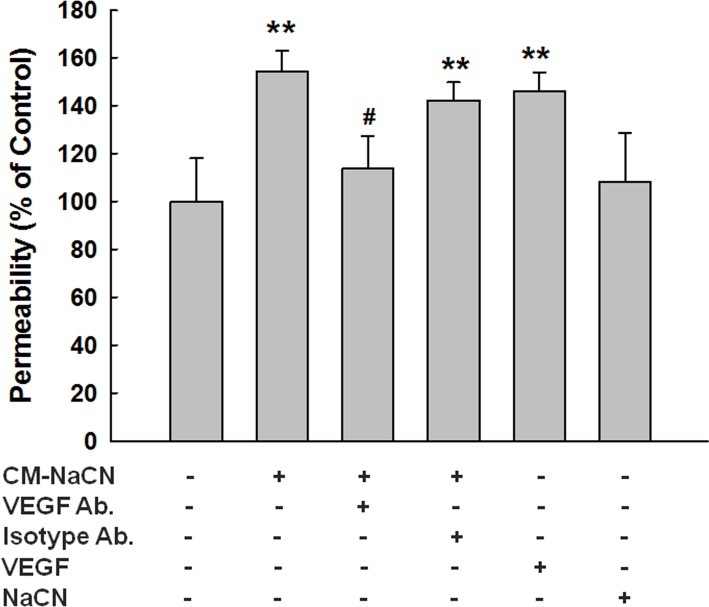
Disruption of endothelial barrier integrity by NaCN-treated pericytes involves VEGF. Conditioned media from pericytes treated with NaCN (2mM) increased endothelial barrier permeability, which was ameliorated in HBMECs pretreated with the VEGF-neutralizing antibody. VEGF (100ng/ml)-treated group was used a positive control. All the data are presented as mean±SD of four individual experiments (n = 4). **p<0.01 vs control group; #p<0.05 vs NaCN-treated group.

The fundamental basis for the BBB is the formation of complex tight junctions between adjacent capillary endothelial cells and interaction of pericytes with these cells. In order to assess whether NaCN-exposed pericytes mediated endothelial barrier breach, human brain microvascular endothelial cells (HBMECs) were exposed to conditioned media from pericytes treated with NaCN and assessed for cell permeability. As shown in Fig 5, HBMECs treated with NaCN-exposed pericyte condition media, demonstrated increased barrier permeability. Exposure of HBMECS to NaCN-exposed pericyte conditioned media that was pretreated with VEGF neutralizing antibody failed to demonstrate endothelial barrier breach, thereby implicating the role of VEGF in NaCN-mediated disruption of BBB. In order to further valid the role of VEGF in endothelial barrier damage, HBMECs were treated with VEGF (100ng/ml) alone. As shown in Fig 5, VEGF exposure resulted in increased endothelial cell permeability. However, exposure of HBMECs to NaCN alone failed to affect the integrity of endothelial cells.

### Disruption of BBB and increased expression of VEGF in the brains of mice with stroke

**Fig 6:**
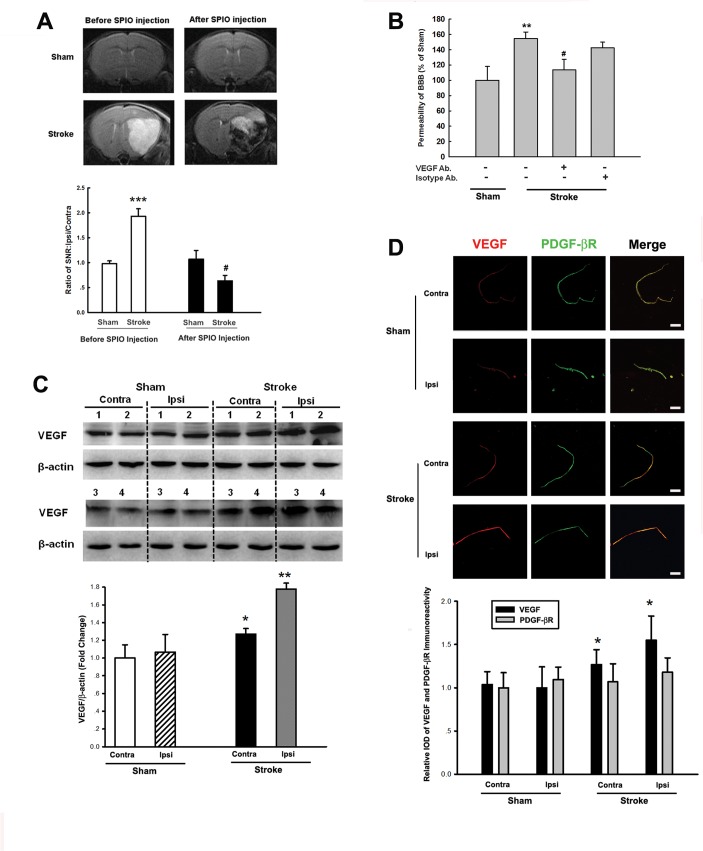
Disrupted BBB and increased expression of VEGF in the brains of stroked mice. **(A)** Representative image of significant signal loss in the lesioned area of the brain on T2-weight images at 24 hours after injection of SPIO in the stroked mice upper panel) and the quantification of signal loss (lower panel). **(B)** Pretreatment of mice with anti-VEGF neutralizing antibody significantly attenuated the increased permeability of BBB in stroke mice. n = 6 per group. All the data are presented as mean±SD. **p<0.01 vs control group; #p<0.05 vs sham group. **(C)** Expression of VEGF in the striatum isolated from sham and stroked mice by western blotting. Representative immunoblots and densitometric analyses of VEGF/β-actin from 4 mice/group are presented. Contra:contralateral; Ipsi:ipsilateral. **(D)** Double immunofluorescence staining specific for VEGF and pericyte marker PDGF-βR in isolated microvessles from sham and stroke mice. VEGF: red; PDGF-βR: green. Scale bar = 100μm. n = 8 per group. All the data are presented as mean±SD. *p<0.05, **p<0.01, ***p<0.001 vs control group; #p<0.05 vs sham group.

For the *in vivo* relevance of our *in vitro* findings, we next examined barrier integrity in the focal stroke model. Briefly, twenty-four hours following surgery to initiate the focal stroke, BBB integrity was examined in the sham and stroke groups of mice using tail vein injection of SPIO particles. As demonstrated in Fig 6A, and as expected, there was a disruption of the BBB integrity in the lesioned area of the brain as shown by the fact that there was significant loss of signal evidenced by T2-weight images at 24 hours post SPIO injection. In order to examine whether anti-VEGF antibody exert potential protection of the BBB integrity, mice were injected with anti-VEGF neutralizing antibody or the IgG isotype control antibody for 2 days before stroke operation. As shown in Fig 6B, anti-VEGF neutralizing antibody significantly attenuated the damage of BBB integrity in stroke mice, but not the IgG isotype control antibody treated group. Further validation of the *in vitro* findings involved assessment of expression of VEGF in the brain homogenates of the contralateral and ipsilateral of brain in sham and stroke groups. As shown in Fig 6C, there was increased expression of VEGF in the brain region in the mice with stroke compared with the sham group. Interestingly, there was increased expression of VEGF in the both contralateral and ipsilateral side of stroke compared with sham group, though there was only a small increase of VEGF expression in the contralateral side as noted. For further validation that the pericytes were the source of increased VEGF, microvessels from stroke versus sham mice were stained for VEGF and platelet-derived growth factor-β receptor (PDGF-βR, a well known pericyte marker) by double immunofluorescent staining. As shown in Fig 6D, in the microvessels from stroke mice, there was increased expression of VEGF co-localized with PDGF-βR compared with sham mice, suggesting thereby that the primary source of increased expression of VEGF was, at least part, from the pericytes, in the stroke model.

## Discussion

Although previous studies have reported the interaction of endothelial cells with astrocytes [19,46], the impact of pericytes on endothelial barrier integrity remains less understood. The role of pericytes in endothelial integrity remains controversial. For example, it has been shown by Nakagawa *et al* that pericytes enhanced the barrier integrity of endothelial cells, however, it has also been reported that pericytes are an important source of matrix metalloproteinase and inflammatory cytokines, and thus contribute to BBB disruption under various pathological CNS conditions [47]. It has been well-recognized that BBB disruption is an early process in ischemic brain injury; however, the contribution of pericytes in this process remains less clear.

In this study, we demonstrated that exposure of pericytes to NaCN as a mimic for ischemic injury, resulted in increased expression of VEGF via activation of tyrosine kinase Src with downstream activation of MAPK and PI3K/Akt pathways, ultimately resulting in translocation of NF-κB into the nucleus. Furthermore, an *in vitro* BBB system consisting of brain microvascular endothelial cells and pericytes was employed to assess the impact of ischemic pericytes on the BBB permeability. Our findings suggested that conditioned media from NaCN-treated pericytes triggered a breach of the BBB, and that this process was mediated, at least, in part, by release of VEGF from NaCN-treated pericytes. Collectively, pericytic VEGF could be one of the attractive therapeutic targets for amelioration of BBB dysfunction in stroke. However, it must be pointed out that BBB function *in viv*o is regulated by several other cell types of microenvironment including astrocyte, microglia and oligodendrocytes in addition to pericytes [48]. Previous study indicated that ischemic condition also induced the expression of VEGF from astrocytes [29], suggesting specific interventions targeting regulation of VEGF expression in astrocytes may be another strategy for protection of BBB. Moreover, this finding was coincident with the previous reports which demonstrated that both astrocytic and pericytic MMP-9 contributed to the BBB damage [48]. BBB disruption during or after stroke attributed to a complex regulatory network, in which pericytes were not the only cell type contributing the BBB dysfunction. Further detailed study about the portion of VEGF from astrocytes or pericytes was needed to determine whether pericyte-derived VEGF explains the major factor in BBB disruption after stroke.

VEGF, one of the key factors induced by hypoxia, exerts a major role in critical disruption of the BBB leading to subsequent edema in the brain during ischemic injury [49,50]. VEGF not only plays critical roles in regulation of endothelial barrier integrity, cell survival and proliferation under physiologic conditions [51], but also is involved in brain disorders including stroke [52]. Up-regulation of VEGF appeared as early as 1hr following the onset of cerebral ischemia [20,53]. Consistent with a recent study that expression of VEGF was up-regulated in astrocytes contributing to increased endothelial permeability [29], we also detected increased expression and release of VEGF in the pericyte conditioned media compared with other cytokine/chemokines. In addition to VEGF, treatment of cells with NaCN also resulted in increase of IL-6 and G-CSF expression (Table 1). Further investigation was needed to elucidate relevance of IL-6 and G-CSF with the endothelial barrier permeability.

Furthermore, exposure of pericytes to NaCN resulted in increased phosphorylation of Src, MAPK and Akt signaling mediators. Inhibition of their activity by using the respective inhibitors significantly abrogated NaCN-mediated induction of VEGF. To our knowledge, this is the first study aimed at exploring the detailed signaling pathway involved in NaCN-mediated induction of VEGF in pericytes. Consistent with the previous studies [41,43,44], activation of Src, MAPKs as well as PI3K pathways were also involved in NaCN-mediated induction of VEGF expression. Our findings are in disagreement with previous reports wherein tissue-type plasminogen activator-mediated induction of VEGF expression involved ERK and p38 MAPKs, but not JNK [54] and another study in which VEGF expression was up-regulated via activation of Akt and ERK MAPK pathways in endothelial progenitor cells [44]. This discrepancy could be attributed likely to the different cell types and cell stimulants used in these two studies.

The transcription factor, NF-kB, has emerged as a major regulatory transcription factor for a number of genes including growth factors such as VEGF [45]. In this study we demonstrated that following NaCN exposure of pericytes, there was rapid translocation of NF-kB into the nucleus. Using pharmacological approach we further demonstrated that activation of MAPKs and PI3K/Akt pathways was upstream of NF-kB. Involvement of NF-kB in NaCN-mediated VEGF induction was validated using a pharmacological approach as demonstrated by the fact that Ikk-2 inhibitor SC514 significantly inhibited NaCN-induced expression of VEGF in pericytes. Our findings are in agreement with a previous report by Omar *et al* identifying the role of NF-kB in expression of VEGF in human umbilical vein endothelial cells [45].

To assess the functional implication(s) of NaCN-induced VEGF in pericytes, we employed an *in vitro* endothelial cell permeability assay. Taking into account that VEGF is a known agent involved in disruption of BBB and since VEGF was one of the key factors that was up-regulated in NaCN-treated pericytes, we examined the role of this factor in endothelial barrier breach. Conditioned media from NaCN-treated pericytes led to increased BBB permeability with the involvement of VEGF as shown by the ability of VEGF neutralizing antibody to block this effect. These findings are in agreement with a recent study implicating the role of VEGF family members in BBB damage [29]. The significance of this study is that it for the first time provides evidence that pericytes can also contribute to BBB damage via stimulation of VEGF expression in the context of stroke. It is likely that VEGF released from the pericytes in response to NaCN exerts its effects by binding to the VEGF receptor expressed in endothelial cells. Binding of VEGF to VEGF receptors could, in turn, activate intracellular signaling leading to barrier breach. Both VEGF and its VEGFR-1 (Flt-1) are expressed in both microvascular endothelial cells and pericytes, however, VEGFR-2 is expressed exclusively in the endothelial cells [55]. Whether VEGFR-2 plays a role in the increased permeability of endothelial barrier is currently under investigation.

Our *in vivo* study lends further credence to our *in vitro* findings as evidenced by the fact that there was indeed increased expression of VEGF in the damaged area from the stroked mice compared with the sham group (Fig 6). Consistent with the previous report that inflammatory biomarkers (VCAM-1, ICAM-1 and P-selectin) were up-regulated within the entire brain after stroke [56], our current study indicated that there was increased expression of VEGF in the both contralateral and ipsilateral side of stroke mice compared with sham group, though there was only a small increase of VEGF expression in the contralateral side as noted. Regarding of the VEGF expression in stroke, our finding was consistent with the previous study that the expression of VEGF was transiently increased in contralateral side of stroke compared with sham control with peak response [57].

**Fig 7:**
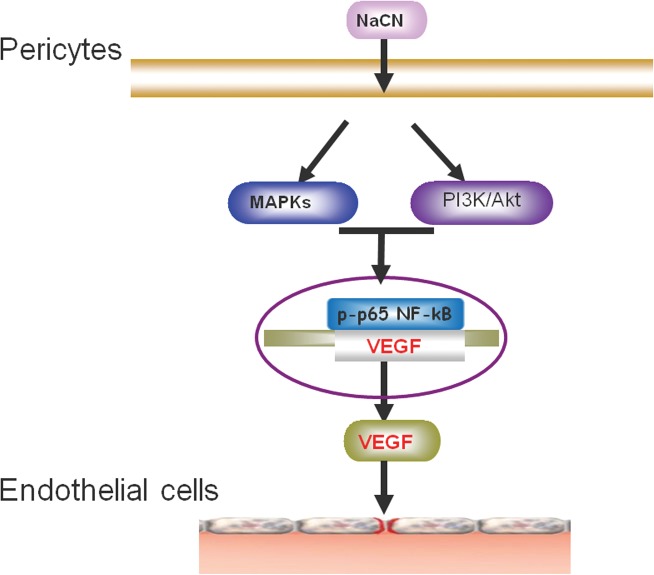
Schematic of the signaling pathways involved in NaCN-mediated induction of VEGF in primary human pericytes. Exposure of pericytes to NaCN resulted in activation of Src, MAPKs and PI3K/Akt signaling pathways, with the downstream activation of the NF-kB transcription factor leading to enhanced expression of VEGF and subsequent increased permeability of endothelial cells.

In conclusion, our findings demonstrate detailed molecular mechanisms underlying increased expression of VEGF mediated by NaCN in primary human pericytes with the involvement of activation of MAPKs and PI3K/Akt and downstream transcription factor NF-kB pathways. The schematic of how pericytes induce VEGF in response to NaCN is summarized in Fig 7. These findings provide insights into the development of potential therapeutic targets for stroke with the role of pericytes in this process.

## References

[1] Zlokovic BV. Cerebrovascular permeability to peptides: manipulations of transport systems at the blood-brain barrier. Pharm Res.1995;12: 1395–1406. 8584471 10.1023/a:1016254514167

[2] Zlokovic BV, Apuzzo ML. Cellular and molecular neurosurgery: pathways from concept to reality—part I: target disorders and concept approaches to gene therapy of the central nervous system. Neurosurgery.1997;40: 789–803. 9092853 10.1097/00006123-199704000-00027

[3] Zlokovic BV, Lipovac MN, Begley DJ, Davson H, Rakic L. Transport of leucine-enkephalin across the blood-brain barrier in the perfused guinea pig brain. J Neurochem.1987;49: 310–315. 3585338 10.1111/j.1471-4159.1987.tb03431.x

[4] Zlokovic BV, Begley DJ, Chain-Eliash DG. Blood-brain barrier permeability to leucine-enkephalin, D-alanine2-D-leucine5-enkephalin and their N-terminal amino acid (tyrosine). Brain Res.1985;336: 125–132. 3891014 10.1016/0006-8993(85)90423-8

[5] Zlokovic BV, Hyman S, McComb JG, Lipovac MN, Tang G, Davson H. Kinetics of arginine-vasopressin uptake at the blood-brain barrier. Biochim Biophys Acta.1990;1025: 191–198. 2364078 10.1016/0005-2736(90)90097-8

[6] Zlokovic BV, Mackic JB, Djuricic B, Davson H. Kinetic analysis of leucine-enkephalin cellular uptake at the luminal side of the blood-brain barrier of an in situ perfused guinea-pig brain. J Neurochem.1989;53: 1333–1340. 2795003 10.1111/j.1471-4159.1989.tb08522.x

[7] Yao Y, Chen ZL, Norris EH, Strickland S. Astrocytic laminin regulates pericyte differentiation and maintains blood brain barrier integrity. Nat Commun.2014;5: 3413 10.1038/ncomms4413 24583950 PMC3992931

[8] Persidsky Y, Ramirez SH, Haorah J, Kanmogne GD. Blood-brain barrier: structural components and function under physiologic and pathologic conditions. J Neuroimmune Pharmacol.2006;1: 223–236. 18040800 10.1007/s11481-006-9025-3

[9] Dore-Duffy P, Katychev A, Wang X, Van Buren E. CNS microvascular pericytes exhibit multipotential stem cell activity. J Cereb Blood Flow Metab.2006;26: 613–624. 16421511 10.1038/sj.jcbfm.9600272

[10] Dohgu S, Takata F, Yamauchi A, Nakagawa S, Egawa T, Naito M, et al Brain pericytes contribute to the induction and up-regulation of blood-brain barrier functions through transforming growth factor-beta production. Brain Res.2005;1038: 208–215. 15757636 10.1016/j.brainres.2005.01.027

[11] Nakagawa S, Deli MA, Kawaguchi H, Shimizudani T, Shimono T, Kittel A, et al A new blood-brain barrier model using primary rat brain endothelial cells, pericytes and astrocytes. Neurochem Int.2009;54: 253–263. 10.1016/j.neuint.2008.12.002 19111869

[12] Takata F, Dohgu S, Yamauchi A, Sumi N, Nakagawa S, Naito M, et al Inhibition of transforming growth factor-beta production in brain pericytes contributes to cyclosporin A-induced dysfunction of the blood-brain barrier. Cell Mol Neurobiol.2007;27: 317–328. 17192829 10.1007/s10571-006-9125-x PMC11881814

[13] Daneman R, Zhou L, Kebede AA, Barres BA. Pericytes are required for blood-brain barrier integrity during embryogenesis. Nature.2010;468: 562–566. 10.1038/nature09513 20944625 PMC3241506

[14] Armulik A, Genove G, Mae M, Nisancioglu MH, Wallgard E, Niaudet C, et al Pericytes regulate the blood-brain barrier. Nature.2010;468: 557–561. 10.1038/nature09522 20944627

[15] Zipser BD, Johanson CE, Gonzalez L, Berzin TM, Tavares R, Hulette CM, et al Microvascular injury and blood-brain barrier leakage in Alzheimer's disease. Neurobiol Aging.2007;28: 977–986. 16782234 10.1016/j.neurobiolaging.2006.05.016

[16] van Vliet EA, da Costa Araujo S, Redeker S, van Schaik R, Aronica E, Gorter JA. Blood-brain barrier leakage may lead to progression of temporal lobe epilepsy. Brain.2007;130: 521–534. 17124188 10.1093/brain/awl318

[17] Wardlaw JM, Doubal F, Armitage P, Chappell F, Carpenter T, Munoz Maniega S, et al Lacunar stroke is associated with diffuse blood-brain barrier dysfunction. Ann Neurol.2009;65: 194–202. 10.1002/ana.21549 19260033

[18] Melgar MA, Rafols J, Gloss D, Diaz FG. Postischemic reperfusion: ultrastructural blood-brain barrier and hemodynamic correlative changes in an awake model of transient forebrain ischemia. Neurosurgery.2005;56: 571–581. 15730583 10.1227/01.neu.0000154702.23664.3d

[19] Abbott NJ, Ronnback L, Hansson E. Astrocyte-endothelial interactions at the blood-brain barrier. Nat Rev Neurosci.2006;7: 41–53. 16371949 10.1038/nrn1824

[20] Abumiya T, Lucero J, Heo JH, Tagaya M, Koziol JA, Copeland BR, et al Activated microvessels express vascular endothelial growth factor and integrin alpha(v)beta3 during focal cerebral ischemia. J Cereb Blood Flow Metab.1999;19: 1038–1050. 10478656 10.1097/00004647-199909000-00012

[21] Argaw AT, Asp L, Zhang J, Navrazhina K, Pham T, Mariani JN, et al Astrocyte-derived VEGF-A drives blood-brain barrier disruption in CNS inflammatory disease. J Clin Invest.2012;122: 2454–2468. 10.1172/JCI60842 22653056 PMC3386814

[22] Bauer AT, Burgers HF, Rabie T, Marti HH. Matrix metalloproteinase-9 mediates hypoxia-induced vascular leakage in the brain via tight junction rearrangement. J Cereb Blood Flow Metab.2010;30: 837–848. 10.1038/jcbfm.2009.248 19997118 PMC2949161

[23] McColl BW, Rose N, Robson FH, Rothwell NJ, Lawrence CB. Increased brain microvascular MMP-9 and incidence of haemorrhagic transformation in obese mice after experimental stroke. J Cereb Blood Flow Metab.2010;30: 267–272. 10.1038/jcbfm.2009.217 19826431 PMC2949124

[24] Takata F, Dohgu S, Matsumoto J, Takahashi H, Machida T, Wakigawa T, et al Brain pericytes among cells constituting the blood-brain barrier are highly sensitive to tumor necrosis factor-alpha, releasing matrix metalloproteinase-9 and migrating in vitro. J Neuroinflammation.2011;8: 106 10.1186/1742-2094-8-106 21867555 PMC3182916

[25] Kovac A, Erickson MA, Banks WA. Brain microvascular pericytes are immunoactive in culture: cytokine, chemokine, nitric oxide, and LRP-1 expression in response to lipopolysaccharide. J Neuroinflammation.2011;8: 139 10.1186/1742-2094-8-139 21995440 PMC3207972

[26] Bell RD, Winkler EA, Sagare AP, Singh I, LaRue B, Deane R, et al Pericytes control key neurovascular functions and neuronal phenotype in the adult brain and during brain aging. Neuron.2010;68: 409–427. 10.1016/j.neuron.2010.09.043 21040844 PMC3056408

[27] Fernandez-Klett F, Potas JR, Hilpert D, Blazej K, Radke J, Huck J, et al Early loss of pericytes and perivascular stromal cell-induced scar formation after stroke. J Cereb Blood Flow Metab.2013;33: 428–439. 10.1038/jcbfm.2012.187 23250106 PMC3587816

[28] Argaw AT, Gurfein BT, Zhang Y, Zameer A, John GR. VEGF-mediated disruption of endothelial CLN-5 promotes blood-brain barrier breakdown. Proc Natl Acad Sci U S A.2009;106: 1977–1982. 10.1073/pnas.0808698106 19174516 PMC2644149

[29] Li YN, Pan R, Qin XJ, Yang WL, Qi Z, Liu W, et al Ischemic neurons activate astrocytes to disrupt endothelial barrier via increasing VEGF expression. J Neurochem.2014;129: 120–129. 10.1111/jnc.12611 24251624 PMC3965617

[30] Arumugam TV, Chan SL, Jo DG, Yilmaz G, Tang SC, Cheng A, et al Gamma secretase-mediated Notch signaling worsens brain damage and functional outcome in ischemic stroke. Nat Med.2006;12: 621–623. 16680150 10.1038/nm1403

[31] Yao H, Kim K, Duan M, Hayashi T, Guo M, Morgello S, et al Cocaine hijacks sigma1 receptor to initiate induction of activated leukocyte cell adhesion molecule: implication for increased monocyte adhesion and migration in the CNS. J Neurosci.2011;31: 5942–5955. 10.1523/JNEUROSCI.5618-10.2011 21508219 PMC3410749

[32] Yao H, Yang Y, Kim KJ, Bethel-Brown C, Gong N, Funa K, et al Molecular mechanisms involving sigma receptor-mediated induction of MCP-1: implication for increased monocyte transmigration. Blood.2010;115: 4951–4962. 10.1182/blood-2010-01-266221 20354174 PMC2890169

[33] Umemura K, Wada K, Uematsu T, Nakashima M. Evaluation of the combination of a tissue-type plasminogen activator, SUN9216, and a thromboxane A2 receptor antagonist, vapiprost, in a rat middle cerebral artery thrombosis model. Stroke.1993;24: 1077–1081. 8322383 10.1161/01.str.24.7.1077

[34] Chen F, Suzuki Y, Nagai N, Jin L, Yu J, Wang H, et al Rodent stroke induced by photochemical occlusion of proximal middle cerebral artery: evolution monitored with MR imaging and histopathology. Eur J Radiol.2007;63: 68–75. 17337149 10.1016/j.ejrad.2007.01.005

[35] Yao H, Ma R, Yang L, Hu G, Chen X, Duan M, et al MiR-9 promotes microglial activation by targeting MCPIP1. Nature communications.2014;5: 4386 10.1038/ncomms5386 25019481 PMC4104446

[36] Yi H, Bai Y, Zhu X, Lin L, Zhao L, Wu X, et al IL-17A Induces MIP-1alpha Expression in Primary Astrocytes via Src/MAPK/PI3K/NF-kB Pathways: Implications for Multiple Sclerosis. Journal of neuroimmune pharmacology: the official journal of the Society on NeuroImmune Pharmacology.2014;9: 629–641. 10.1007/s11481-014-9553-1 24989845

[37] Yao H, Duan M, Hu G, Buch S. Platelet-derived growth factor B chain is a novel target gene of cocaine-mediated Notch1 signaling: implications for HIV-associated neurological disorders. J Neurosci.2011;31: 12449–12454. 10.1523/JNEUROSCI.2330-11.2011 21880906 PMC3283138

[38] Liu DF, Qian C, An YL, Chang D, Ju SH, Teng GJ. Magnetic resonance imaging of post-ischemic blood-brain barrier damage with PEGylated iron oxide nanoparticles. Nanoscale.2014;6: 15161–15167. 10.1039/c4nr03942d 25374303

[39] Zhang Y, Fan S, Yao Y, Ding J, Wang Y, Zhao Z, et al In Vivo Near-Infrared Imaging of Fibrin Deposition in Thromboembolic Stroke in Mice. PLoS ONE.2012;7: e30262 10.1371/journal.pone.0030262 22272319 PMC3260250

[40] Rose K, Ouellette Y, Bolon M, Tyml K. Hypoxia/reoxygenation reduces microvascular endothelial cell coupling by a tyrosine and MAP kinase dependent pathway. J Cell Physiol.2005;204: 131–138. 15672421 10.1002/jcp.20283

[41] Zan L, Zhang X, Xi Y, Wu H, Song Y, Teng G, et al Src regulates angiogenic factors and vascular permeability after focal cerebral ischemia-reperfusion. Neuroscience.2014;262: 118–128. 10.1016/j.neuroscience.2013.12.060 24412374 PMC3943922

[42] Hernandez-Hernandez OT, Gonzalez-Garcia TK, Camacho-Arroyo I. Progesterone receptor and SRC-1 participate in the regulation of VEGF, EGFR and Cyclin D1 expression in human astrocytoma cell lines. J Steroid Biochem Mol Biol.2012;132: 127–134. 10.1016/j.jsbmb.2012.04.005 22542550

[43] Huang YH, Yang HY, Hsu YF, Chiu PT, Ou G, Hsu MJ. Src contributes to IL6-induced vascular endothelial growth factor-C expression in lymphatic endothelial cells. Angiogenesis.2014;17: 407–418. 10.1007/s10456-013-9386-1 24048742

[44] Tang Y, Vater C, Jacobi A, Liebers C, Zou X, Stiehler M. Salidroside exerts angiogenic and cytoprotective effects on human bone marrow-derived endothelial progenitor cells via Akt/mTOR/p70S6K and MAPK signalling pathways. Br J Pharmacol.2014;171: 2440–2456. 10.1111/bph.12611 24471788 PMC3997282

[45] Omar HA, Arafa el SA, Salama SA, Arab HH, Wu CH, Weng JR. OSU-A9 inhibits angiogenesis in human umbilical vein endothelial cells via disrupting Akt-NF-kappaB and MAPK signaling pathways. Toxicol Appl Pharmacol.2013;272: 616–624. 10.1016/j.taap.2013.07.014 23921148

[46] Meyer J, Rauh J, Galla HJ. The susceptibility of cerebral endothelial cells to astroglial induction of blood-brain barrier enzymes depends on their proliferative state. J Neurochem.1991;57: 1971–1977. 1719132 10.1111/j.1471-4159.1991.tb06411.x

[47] Thanabalasundaram G, Pieper C, Lischper M, Galla HJ. Regulation of the blood-brain barrier integrity by pericytes via matrix metalloproteinases mediated activation of vascular endothelial growth factor in vitro. Brain Res.2010;1347: 1–10. 10.1016/j.brainres.2010.05.096 20553880

[48] Neuhaus W, Gaiser F, Mahringer A, Franz J, Riethmuller C, Forster C. The pivotal role of astrocytes in an in vitro stroke model of the blood-brain barrier. Front Cell Neurosci.2014;8: 352 10.3389/fncel.2014.00352 25389390 PMC4211409

[49] Engelhardt S, Patkar S, Ogunshola OO. Cell-specific blood-brain barrier regulation in health and disease: a focus on hypoxia. Br J Pharmacol.2014;171: 1210–1230. 10.1111/bph.12489 24641185 PMC3952799

[50] Zhang ZG, Zhang L, Jiang Q, Zhang R, Davies K, Powers C, et al VEGF enhances angiogenesis and promotes blood-brain barrier leakage in the ischemic brain. J Clin Invest.2000;106: 829–838. 11018070 10.1172/JCI9369 PMC517814

[51] Thanabalasundaram G, Schneidewind J, Pieper C, Galla HJ. The impact of pericytes on the blood-brain barrier integrity depends critically on the pericyte differentiation stage. Int J Biochem Cell Biol.2011;43: 1284–1293. 10.1016/j.biocel.2011.05.002 21601005

[52] Shibuya M. Brain angiogenesis in developmental and pathological processes: therapeutic aspects of vascular endothelial growth factor. FEBS J.2009;276: 4636–4643. 10.1111/j.1742-4658.2009.07175.x 19664071

[53] Plate KH, Beck H, Danner S, Allegrini PR, Wiessner C. Cell type specific upregulation of vascular endothelial growth factor in an MCA-occlusion model of cerebral infarct. J Neuropathol Exp Neurol.1999;58: 654–666. 10374756 10.1097/00005072-199906000-00010

[54] Duan P, Ni C. t-PA stimulates VEGF expression in endothelial cells via ERK2/p38 signaling pathways. Pharmazie.2014;69: 70–75. 24601228

[55] Witmer AN, van Blijswijk BC, van Noorden CJ, Vrensen GF, Schlingemann RO. In vivo angiogenic phenotype of endothelial cells and pericytes induced by vascular endothelial growth factor-A. J Histochem Cytochem.2004;52: 39–52. 14688216 10.1177/002215540405200105

[56] Gauberti M, Montagne A, Marcos-Contreras OA, Le Behot A, Maubert E, Vivien D. Ultra-sensitive molecular MRI of vascular cell adhesion molecule-1 reveals a dynamic inflammatory penumbra after strokes. Stroke.2013;44: 1988–1996. 10.1161/STROKEAHA.111.000544 23743972

[57] Cheung WM, Chen SF, Nian GM, Lin TN. Induction of angiogenesis related genes in the contralateral cortex with a rat three-vessel occlusion model. Chin J Physiol.2000;43: 119–124. 11132088

